# Inappropriate Diagnosis of Pneumonia Among Hospitalized Adults

**DOI:** 10.1001/jamainternmed.2024.0077

**Published:** 2024-03-25

**Authors:** Ashwin B. Gupta, Scott A. Flanders, Lindsay A. Petty, Tejal N. Gandhi, Michael S. Pulia, Jennifer K. Horowitz, David Ratz, Steven J. Bernstein, Anurag N. Malani, Payal K. Patel, Timothy P. Hofer, Tanima Basu, Vineet Chopra, Valerie M. Vaughn

**Affiliations:** 1 Medicine Service, VA Ann Arbor Healthcare System, Ann Arbor, Michigan; 2 Division of Hospital Medicine, Department of Internal Medicine, University of Michigan Medical School, Ann Arbor; 3 Division of Infectious Diseases, Department of Internal Medicine, University of Michigan Medical School, Ann Arbor; 4 Department of Emergency Medicine, University of Wisconsin School of Medicine and Public Health, Madison; 5 Veterans Affairs Center for Clinical Management Research, Ann Arbor, Michigan; 6 Division of General Medicine, Department of Internal Medicine, University of Michigan Medical School, Ann Arbor; 7 Section of Infectious Diseases, Trinity Health Michigan, Ann Arbor; 8 Intermountain Healthcare, Salt Lake City, Utah; 9 Department of Medicine, University of Colorado School of Medicine, Aurora; 10 Division of General Internal Medicine, Department of Internal Medicine, University of Utah School of Medicine, Salt Lake City; 11 Division of Health System Innovation & Research, Department of Population Health Sciences, University of Utah School of Medicine, Salt Lake City

## Abstract

**Importance:**

Little is known about incidence of, risk factors for, and harms associated with inappropriate diagnosis of community-acquired pneumonia (CAP).

**Objective:**

To characterize inappropriate diagnosis of CAP in hospitalized patients.

**Design, Setting, and Participants:**

This prospective cohort study, including medical record review and patient telephone calls, took place across 48 Michigan hospitals. Trained abstractors retrospectively assessed hospitalized patients treated for CAP between July 1, 2017, and March 31, 2020. Patients were eligible for inclusion if they were adults admitted to general care with a discharge diagnostic code of pneumonia who received antibiotics on day 1 or 2 of hospitalization. Data were analyzed from February to December 2023.

**Main Outcomes and Measures:**

Inappropriate diagnosis of CAP was defined using a National Quality Forum–endorsed metric as CAP-directed antibiotic therapy in patients with fewer than 2 signs or symptoms of CAP or negative chest imaging. Risk factors for inappropriate diagnosis were assessed and, for those inappropriately diagnosed, 30-day composite outcomes (mortality, readmission, emergency department visit, *Clostridioides difficile *infection, and antibiotic-associated adverse events) were documented and stratified by full course (>3 days) vs brief (≤3 days) antibiotic treatment using generalized estimating equation models adjusting for confounders and propensity for treatment.

**Results:**

Of the 17 290 hospitalized patients treated for CAP, 2079 (12.0%) met criteria for inappropriate diagnosis (median [IQR] age, 71.8 [60.1-82.8] years; 1045 [50.3%] female), of whom 1821 (87.6%) received full antibiotic courses. Compared with patients with CAP, patients inappropriately diagnosed were older (adjusted odds ratio [AOR], 1.08; 95% CI, 1.05-1.11 per decade) and more likely to have dementia (AOR, 1.79; 95% CI, 1.55-2.08) or altered mental status on presentation (AOR, 1.75; 95% CI, 1.39-2.19). Among those inappropriately diagnosed, 30-day composite outcomes for full vs brief treatment did not differ (25.8% vs 25.6%; AOR, 0.98; 95% CI, 0.79-1.23). Full vs brief duration of antibiotic treatment among patients was associated with antibiotic-associated adverse events (31 of 1821 [2.1%] vs 1 of 258 [0.4%]; *P* = .03).

**Conclusions and Relevance:**

In this cohort study, inappropriate diagnosis of CAP among hospitalized adults was common, particularly among older adults, those with dementia, and those presenting with altered mental status. Full-course antibiotic treatment of those inappropriately diagnosed with CAP may be harmful.

## Key Points

**Question:**

In hospitalized patients diagnosed with pneumonia, what is the incidence of and factors associated with inappropriate diagnosis?

**Findings:**

In this cohort study of 17 290 hospitalized adults treated for pneumonia in 48 Michigan hospitals, 12.0% were inappropriately diagnosed. Older patients, those with dementia, and those presenting with altered mental status had the highest risk of being inappropriately diagnosed, and for those inappropriately diagnosed, receipt of a full antibiotic duration was associated with antibiotic-associated adverse events.

**Meaning:**

Inappropriate diagnosis of pneumonia among hospitalized adults is common, particularly among older adults with geriatric syndromes, and may be harmful.

## Teaser

This cohort study characterizes inappropriate diagnosis of community-acquired pneumonia among hospitalized patients in 48 Michigan hospitals.

## Introduction

Lower respiratory tract infection, including community-acquired pneumonia (CAP), is the fourth most common cause of medical hospitalization^1^ and most common infectious cause of hospitalization in the US.^2,3^ While many hospitalized patients treated for pneumonia have an infection, inaccurate or inappropriate diagnosis of pneumonia (ie, pneumonia diagnosis when pneumonia is not present) is common.^4,5^

While some inappropriate diagnosis of CAP is unavoidable due to diagnostic uncertainty when patients are first hospitalized, many patients remain inappropriately diagnosed even on hospital discharge.^6^ Inappropriate diagnosis of CAP may harm patients through delayed recognition and treatment of acute (eg, exacerbations of congestive heart failure^7^), chronic (eg, pulmonary cancer^8^), or novel diagnoses (eg, pulmonary cancer^8^) and may lead to unnecessary antibiotic use,^9,10^ adverse effects, and antibiotic resistance.^11,12,13^

Accurately quantifying the proportion of patients treated for CAP who are inappropriately diagnosed has been challenging due to lack of validated definitions. In 2022, we devised a metric to quantify inappropriate diagnosis of CAP that was validated and subsequently endorsed by the National Quality Forum (NQF).^14,15,16^ Herein, we apply this metric to a cohort of hospitalized patients treated for CAP in 48 Michigan hospitals to understand epidemiology and outcomes associated with inappropriate CAP diagnosis.

## Methods

### Study Setting and Design

The Michigan Hospital Medicine Safety Consortium (HMS) is a multi-institutional collaborative quality initiative developed to improve care for hospitalized patients. As of January 2023, 69 of 92 (75%) noncritical access, nonfederal hospitals in Michigan participate in HMS and share data on antibiotic use. This study includes 48 hospitals participating during the entire study period.

Because the purpose of HMS is to improve the quality of current care practices, this study received a status designation of not regulated by the University of Michigan Medical School’s institutional review board, which also waived the requirement for informed consent. We followed Strengthening the Reporting of Observational Studies in Epidemiology (STROBE) reporting guidelines.^17^

### Patient Sampling and Selection

As in prior HMS studies,^10,18^ trained abstractors retrospectively assessed patients hospitalized and treated for pneumonia between July 1, 2017, and March 31, 2020. Patients were eligible for inclusion if they were adults admitted to general care with a discharge diagnostic code of pneumonia^19^ who received antibiotics on day 1 or 2 of hospitalization (to specify community-onset infection; see eligible *International Statistical Classification of Diseases and Related Health Problems, Tenth Revision, *codes in the eAppendix in Supplement 1). Patients who had documentation of treatment for an additional infection unrelated to pneumonia, were severely immunocompromised, were pregnant, were admitted for comfort measures, or who left against medical advice were ineligible. Abstractors screened consecutive patients via medical record review 30 days after discharge and included the first eligible patient daily. We excluded patients who received care in an intensive care unit or who received ventilation at any point during the hospitalization, were admitted to hospitals with fewer than 10 qualifying patients, had unknown or missing antibiotic treatment data, received more than 14 days of antibiotic treatment (ie, likely to have an infectious complication), or met all eligibility criteria but had a documented chronic obstructive pulmonary disease (COPD) exacerbation treated with azithromycin or doxycycline alone (ie, no additional pneumonia treatment). See the eAppendix in Supplement 1 for detailed definitions and protocols.

### Data Collection

HMS’s data collection and quality assurance procedures have been previously described.^10,18^ Briefly, trained abstractors entered data from the medical record into a central deidentified repository. Patient data, including demographics, comorbid and concurrent conditions, laboratory studies, vital signs, antibiotic type and duration, and outcomes, were collected from 90 days preceding hospitalization through 30 days following hospitalization. To enable assessment of inappropriate diagnosis, abstractors abstracted (1) any signs (eg, leukocytosis) or symptoms (eg, new/increased cough, sputum production, dyspnea) potentially associated with pneumonia documented in clinician and nursing notes through hospital day 3, including documentation associated with the symptom (eg, “patient coughing,” “family-reported increased cough”) (Table 1 and eAppendix in Supplement 1); (2) radiographic findings (eg, pulmonary consolidation) from chest imaging reports through day 4 of the hospitalization, including details (eg, single lobe, multifocal), and markers of uncertainty (eg, abstraction tool in the eAppendix in Supplement 1); and (3) antibiotic administration, including in the emergency department or prescribed at hospital discharge.

**Table 1.** Signs, Symptoms, and Radiographic Findings in Patients Meeting Criteria for Community-Acquired Pneumonia (CAP) vs Those Inappropriately Diagnosed

Sign, symptom, or radiographic finding	No. (%)	
	Meeting criteria for CAP (n = 15 211)	Inappropriate diagnosis of CAP (n = 2079)
Respiratory symptoms (on day 1 or 2 of hospitalization)		
New or increased cough	13 152 (86.5)	1369 (65.8)
Change/increase in sputum production	7863 (51.7)	795 (38.2)
New or increased dyspnea^a^	12 132 (79.8)	1328 (63.9)
Respiratory signs (on day 1 or 2 of hospitalization)		
Hypoxemia (oxygen saturation <90% or Pao_2_ <60 mm Hg)	5053 (33.2)	448 (21.5)
Auscultatory findings (eg, egophony, rales)	8912 (58.6)	886 (42.6)
Tachypnea (respiratory rate >20 breaths/min)^a^	10 476 (68.9)	1158 (55.7)
Infectious signs (on day 1 or 2 of hospitalization)		
Temperature >38 °C or ≤36 °C	9131 (60.0)	973 (46.8)
Temperature >38 °C	6779 (44.6)	706 (34.4)
Temperature ≤36 °C	6337 (41.7)	660 (31.7)
Leukocyte count <4000/μL, >10 000/μL, or >15% bands	10 399 (68.4)	1122 (54.0)
Radiologic findings (from any chest imaging in first 3 d of hospitalization)		
Air space density/opacity/disease/consolidation/infiltrate	14 486 (95.2)	480 (23.1)
Aspiration/postobstructive/necrotizing/pneumonia	8362 (55.0)	251 (12.1)
Cavitation	120 (0.8)	2 (0.1)
Ground glass	57 (0.4)	2 (0.1)
Loculations	138 (0.9)	4 (0.2)
Rule out pneumonia/infection	1924 (12.6)	47 (2.3)
Pleural effusion	785 (5.2)	37 (1.8)
Mass	75 (0.5)	3 (0.1)
Tree in bud	4 (<0.1)	0

Abbreviation: Pao_2_, partial pressure of arterial oxygen.

SI conversion factors: To convert Pao_2_ to kilopascals, multiply by 0.133; leukocytes to ×10^9^/L, multiply by 0.001.

^a^ The presence of either new or increased dyspnea or tachypnea qualifies as a single sign or symptom of CAP.

To assess associations between antibiotic use and patient outcomes, abstractors collected data from the medical record through 30 days postdischarge. Outcomes included 30-day mortality, readmission, emergency department visit, *Clostridioides difficile* infection, and/or in-hospital antibiotic-associated adverse events documented by a physician. To reduce risk of missing important outcomes, patients were also called 30 days postdischarge to obtain additional outcome data. Patients discharged to inpatient hospice, an extended care facility, or prison were ineligible for follow-up telephone calls.

### Primary and Secondary Outcomes

The primary outcome of interest was inappropriate diagnosis of CAP. To determine this, we used an NQF-endorsed definition of inappropriate diagnosis of CAP metric, which defines inappropriate diagnosis of CAP as any antibiotic treatment of CAP in a patient with fewer than 2 signs or symptoms of pneumonia or who lacked radiographic findings consistent with pneumonia (eAppendix in Supplement 1).^14,15,16^ This metric underwent substantial validity and reliability testing, including comparison with physician medical record review and evaluation by a national expert panel. In contrast with the NQF definition, we considered patients not meeting criteria for pneumonia who had bacteremia (n = 25) or positive streptococcal (n = 17) or legionella (n = 10) urine antigen tests to be appropriately diagnosed (2 patients had multiple positive tests).

The secondary outcomes were a composite of 30-day patient outcomes including 30-day all-cause postdischarge mortality, hospital readmission, emergency department visit, *C difficile* infection, and/or physician-documented antibiotic-associated adverse event. Mortality, readmission, emergency department visit, and *C difficile* infection included events abstracted from the medical records and patient-reported events obtained from the 30-day postdischarge telephone call. Outcomes were also assessed independently. To assess the association of antibiotic treatment and patient outcomes in patients inappropriately diagnosed, we compared outcomes in patients who received full (>3 days) vs brief (≤3 days) empirical antibiotic treatment. We used 3 days as a cutoff because multiple guidelines recommend reassessing antibiotic necessity in hospitalized patients treated empirically with antibiotics and stopping antibiotics within 48 to 72 hours in the absence of evidence of infection.^20,21^

### Statistical Analysis

Descriptive statistics were used to describe prevalence of inappropriate diagnosis of CAP by hospital, variation of inappropriate diagnosis across hospitals, and characteristics of patients inappropriately diagnosed vs those meeting criteria for CAP. To determine patient characteristics associated with inappropriate diagnosis of CAP, we first compared characteristics of patients with inappropriate diagnosis of CAP vs those meeting criteria for CAP using χ^2^ tests for categorical variables or Wilcoxon rank-sum tests for continuous variables. Next, we created a multivariable logit generalized estimating equation (GEE) model with exchangeable correlation structure to account for the clustering of patients within hospitals. To identify variables retained in the final model, we used a stepwise selection procedure based on the Schwarz criterion,^22^ starting with all variables with *P* < .10 in the bivariable analysis. Variables having collinearity (ie, variables between which a strong correlation exists, making it difficult or impossible to estimate their individual regression coefficients reliably [eg, Pneumonia Severity Index and age, altered mental status]) were removed. Variables known to be associated with diagnosis (eg, age, home oxygen) were forced into the final model.

To characterize the associations of antibiotic treatment with 30-day patient outcomes, we first calculated unadjusted odds ratios (ORs) comparing outcomes between full vs brief empirical antibiotic treatment. Next, we identified characteristics of full vs brief empirical antibiotic treatment among patients inappropriately diagnosed with CAP. Finally, we used logit GEE models with exchangeable correlation to account for clustering of patients within hospitals to compare the composite 30-day postdischarge patient outcome (and each individual 30-day postdischarge outcome) in patients inappropriately diagnosed with CAP who received full vs brief empirical antibiotic therapy. Final GEE models were adjusted for baseline covariates known to be associated with outcomes or found to be associated with full vs brief empirical treatment (covariates listed in Table 2). Because missing data were minimal among patients (insurance, 3.3%; race, 0.3%; and body mass index, 1.0%), we did not impute missing variables. Results of all multivariable models were expressed as adjusted odds ratios (AORs) with 95% CIs, and *P* < .05 was considered statistically significant. Analyses were performed in SAS, version 9.4 (SAS Institute). Because disparities related to patient demographics may be present, we report data on sex and race and ethnicity obtained from the medical records (eAppendix in Supplement 1).

**Table 2.** 30-Day Adverse Outcomes Among Patients Inappropriately Diagnosed With Community-Acquired Pneumonia (CAP) Treated With Full vs Brief Empirical Antibiotic Therapy^a^

30-d Postdischarge outcomes	No. (%)			Unadjusted odds ratio (95% CI)	*P* value	Adjusted odds ratio (95% CI)	*P* value
	Inappropriate diagnosis of CAP (n = 2079)	Antibiotic duration^b^					
		Full (n = 1821)	Brief (n = 258)				
Mortality^c^	69 (3.3)	61 (3.3)	8 (3.1)	1.00 (0.53-1.88)	.99	1.00 (0.46-2.16)	.99
Readmissions^c^	294 (14.1)	258 (14.2)	36 (14.0)	1.00 (0.74-1.34)	.98	0.99 (0.71-1.37)	.94
Emergency department visits^c^	219 (10.5)	188 (10.3)	31 (12.0)	0.82 (0.58-1.15)	.25	0.85 (0.57-1.26)	.42
*Clostridioides difficile* infection^d^	11 (0.5)	10 (0.5)	1 (0.4)	1.62 (0.11-23.75)	.72	1.39 (0.05-36.74)	.85
Physician-documented antibiotic-associated adverse event^e^	39 (1.9)	38 (2.1)	1 (0.4)	6.26 (1.15-34.02)	.03	7.23 (1.18-44.35)	.03
Composite event^c^^,^^f^	536 (25.8)	470 (25.8)	66 (25.6)	0.98 (0.79-1.23)	.89	1.00 (0.78-1.29)	.99

^a^ Multivariable models adjusted for clustering and propensity for treatment.

^b^ A full antibiotic course was defined as more than 3 days of therapy, and a brief empirical course was defined as 3 or fewer days of therapy. An odds ratio higher than 1 indicates higher adverse events among patients who received a full course of antibiotic therapy.

^c^ Adjusted for age; length of stay; Charlson Comorbidity Index score; hospitalization in the 90 days preceding current admission; admission from nursing home; discharge to long-term acute care, skilled nursing facility, or rehabilitation facility; Medicaid insurance; Pneumonia Severity Index; and congestive heart failure exacerbation, chronic obstructive lung disease exacerbation, and hemodialysis.

^d^ Adjusted for age, antibiotic use in the prior 90 days, Charlson Comorbidity Index score, hospitalization in the 90 days preceding current admission, admission from nursing home, proton pump inhibitor therapy, tube feeding, length of stay, and chronic obstructive lung disease exacerbation.

^e^ Adjusted for age, Charlson Comorbidity Index score, sex, and chronic obstructive lung disease exacerbation.

^f^ Composite adverse events include 30-day postdischarge mortality, 30-day postdischarge readmissions, 30-day postdischarge emergency department visits, *Clostridioides difficile* infection, and physician-documented antibiotic-associated adverse event.

## Results

### Inappropriate Diagnosis of CAP

**Figure 1.**
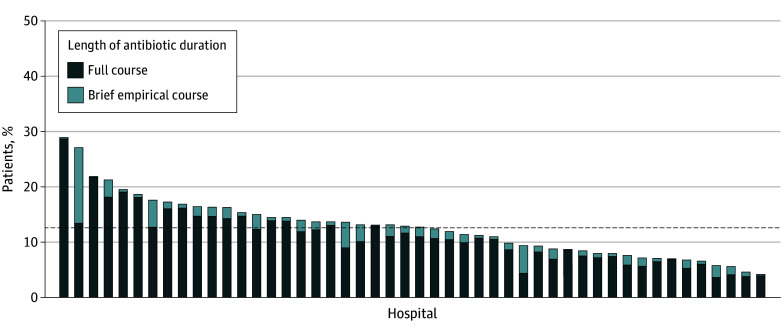
Patients Treated for Community-Acquired Pneumonia Who Were Inappropriately Diagnosed A full course of antibiotic treatment was defined as more than 3 days of therapy, and a brief empirical course was defined as 3 or fewer days of therapy. Each bar represents a hospital (N = 48), and the dashed line indicates the mean proportion of inappropriate diagnosis across all hospitals in the collaborative over the study period.

Between July 1, 2017, and March 1, 2020, 17 290 patients treated for pneumonia were included from 48 Michigan hospitals (eTables 1 and 2 in Supplement 1); 2079 patients (12.0%) met NQF criteria for inappropriate diagnosis. The mean (SD) percentage of patients treated for CAP who were inappropriately diagnosed varied by hospital (12.8% [5.4%]), with 30 of 48 hospitals (62.5%) inappropriately diagnosing 10% or more of patients with CAP (Figure 1).

**Figure 2.**
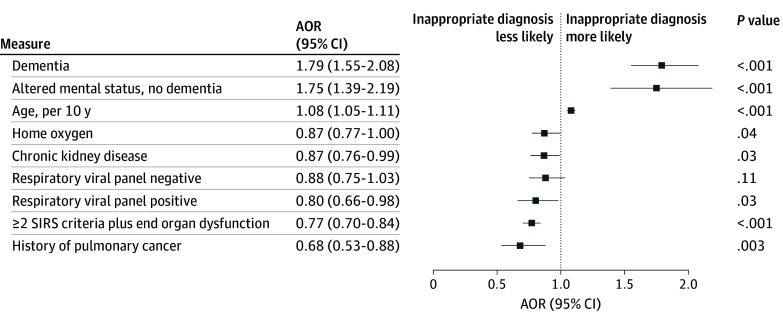
Multivariable Model of Characteristics Associated With Inappropriate vs Appropriate Diagnosis of Community-Acquired Pneumonia Respiratory viral panel (both negative and positive) was compared with no test or missing data, and 2 or more systemic inflammatory response syndrome (SIRS) criteria plus end organ dysfunction was compared with fewer than 2 SIRS criteria. AOR indicates adjusted odds ratio.

Of the 2079 patients inappropriately diagnosed with CAP, 1531 (73.6%) lacked radiographic criteria, 507 (24.4%) had fewer than 2 signs or symptoms of pneumonia, and 41 (2.0%) met neither criterion. Chest computed tomography was obtained in 42.1% of all patients. Like patients with CAP, those inappropriately diagnosed most often presented with dyspnea and/or cough (Table 1). There was no difference in median length of stay for patients meeting vs not meeting criteria for CAP. On bivariable analysis, compared with patients with CAP, patients inappropriately diagnosed with CAP were older (eg, ≥75 years old) and more likely to have public insurance, present with altered mental status, have decreased mobility (eg, bedridden, wheelchair bound) on admission, or have had an inpatient hospitalization within the previous 90 days (Table 3). Regardless of living arrangement on admission (eg, home vs skilled nursing facility), patients inappropriately diagnosed with CAP were more often discharged to a skilled nursing facility. On multivariable analysis, compared with patients with CAP, inappropriately diagnosed patients were older (AOR, 1.08; 95% CI, 1.05-1.11; *P* < .001 per decade) and more likely to have dementia (AOR, 1.79; 95% CI, 1.55-2.08; *P* < .001) or present with altered mental status without dementia (AOR, 1.75; 95% CI, 1.39-2.19; *P* < .001) (Figure 2).

**Table 3.** Characteristics of Patients Treated for Community-Acquired Pneumonia (CAP) by Appropriateness of Diagnosis, Bivariable Analysis

Characteristic	No. (%)			*P* value
	Total (N = 17 290)	Meeting criteria for CAP (n = 15 211)	Inappropriate diagnosis of CAP (n = 2079)	
Age, y				
Median (IQR)	69.8 (58.2-80.8)	69.5 (57.9-80.6)	71.8 (60.1-82.8)	<.001
<65	6805 (39.4)	6090 (40.0)	715 (34.4)	<.001
65-74	3853 (22.3)	3390 (22.3)	463 (22.3)	
≥75	6632 (38.4)	5731 (37.7)	901 (43.3)	
BMI, median (IQR)	27.45 (23.12-33.15)	27.46 (23.12-33.20)	27.33 (23.10-32.81)	.26
Sex				.95
Female	8679 (50.2)	7634 (50.2)	1045 (50.3)	
Male	8611 (49.8)	7577 (49.8)	1034 (49.7)	
Race				.16
Black	3612 (21.0)	3144 (20.7)	468 (22.6)	
White	13 008 (75.5)	11 474 (75.7)	1534 (74.0)	
Other^a^	610 (3.5)	539 (3.6)	71 (3.4)	
Ethnicity				.08
Hispanic	299 (1.7)	253 (1.7)	46 (2.2)	
Non-Hispanic	14 101 (81.6)	12 435 (81.8)	1666 (80.1)	
Unknown	2890 (16.7)	2523 (16.6)	367 (17.7)	
Insurance type				.01
Commercial	4998 (29.9)	4438 (30.2)	560 (27.7)	
Public	11 462 (68.6)	10 019 (68.2)	1443 (71.3)	
Self-pay	252 (1.5)	230 (1.6)	22 (1.1)	
Pneumonia Severity Index, median (IQR)	97.4 (74.2-122.6)	97.6 (74.1-123.2)	96.0 (74.4-119.3)	.03
qSOFA score				
Mean (SD)	0.80 (0.61)	0.81 (0.61)	0.72 (0.64)	<.001
≥2	1670 (9.7)	1480 (9.7)	190 (9.1)	.39
<2	15 620 (90.3)	13 731 (90.3)	1889 (90.9)	
SIRS criteria ≥2 and end organ dysfunction	5821 (33.7)	5214 (34.3)	607 (29.2)	<.001
CHF exacerbation	1403 (8.1)	1251 (8.2)	152 (7.3)	.15
COPD exacerbation	4316 (25.0)	3764 (24.7)	552 (26.6)	.07
Altered mental status	1303 (7.5)	1080 (7.1)	223 (10.7)	<.001
Altered mental status without dementia	867 (5.0)	733 (4.8)	134 (6.4)	.001
Comorbidities				
Charlson Comorbidity Index, median (IQR)	3.0 (1.0-4.0)	3.0 (1.0-5.0)	3.0 (1.0-4.0)	.08
Influenza	611 (3.5)	544 (3.6)	67 (3.2)	.41
CHF	4704 (27.2)	4162 (27.4)	542 (26.1)	.21
COPD	7606 (44.0)	6702 (44.1)	904 (43.5)	.62
Diabetes	5276 (30.5)	4602 (30.3)	674 (32.4)	.04
Dementia	1725 (10.0)	1396 (9.2)	329 (15.8)	<.001
Chronic kidney disease	4960 (28.7)	4399 (28.9)	561 (27.0)	.07
Any cancer^b^	3988 (23.1)	3552 (23.4)	436 (21.0)	.02
Lung cancer	925 (5.3)	846 (5.6)	79 (3.8)	<.001
Current or former smoker	11 467 (66.3)	10 136 (66.6)	1331 (64.0)	.02
Home oxygen	2747 (15.9)	2446 (16.1)	301 (14.5)	.06
Immunosuppression^c^	1188 (6.9)	1072 (7.0)	116 (5.6)	.01
History of MRSA infection	50 (0.3)	45 (0.3)	5 (0.2)	.66
Procalcitonin obtained	4165 (24.1)	3728 (24.5)	437 (21.0)	<.001
Procalcitonin range, ng/mL^d^				.01
0-0.1	1726 (41.4)	1514 (40.6)	212 (48.5)	
>0.1-0.25	879 (21.1)	790 (21.2)	89 (20.4)	
>0.25-0.5	532 (12.8)	484 (13.0)	48 (11.0)	
>0.5	1028 (24.7)	940 (25.2)	88 (20.1)	
Respiratory panel^e^				<.001
Positive	571 (3.3)	524 (3.4)	47 (2.3)	
Negative	2926 (16.9)	2635 (17.3)	291 (14.0)	
No test/missing	13 793 (79.8)	12 052 (79.2)	1741 (83.7)	
Antibiotics within the past 90 d	3817 (22.1)	3352 (22.0)	465 (22.4)	.73
Hospital admission in prior 90 d	4118 (23.8)	3583 (23.6)	535 (25.7)	.03
Admitted from outside skilled nursing facility	591 (3.4)	505 (3.3)	86 (4.1)	.055
Admission from home and discharged to outside skilled nursing facility	1774 (10.3)	1523 (10.0)	251 (12.1)	.004
Functional status on admission				
Bedridden	430 (2.5)	348 (2.3)	82 (3.9)	<.001
Wheelchair bound	746 (4.3)	635 (4.2)	111 (5.3)	.01
Discharged to outside facility	3157 (18.3)	2687 (17.7)	470 (22.6)	<.001
Skilled nursing facility	1745 (10.1)	1453 (9.6)	292 (14.0)	<.001
Subacute rehabilitation	718 (4.2)	638 (4.2)	80 (3.8)	.46
Long-term care facility	45 (0.3)	40 (0.3)	5 (0.2)	.85
Acute rehabilitation facility	103 (0.6)	86 (0.6)	17 (0.8)	.16
Antibiotic duration, median (IQR), d				
Total duration	7 (6-9)	7 (6-9)	7 (5-9)	<.001
Inpatient duration	4 (3-5)	4 (3-5)	4 (3-5)	<.001
Discharge duration	3 (0-5)	3 (0-5)	3 (0-5)	<.001
Length of stay, median (IQR), d	5 (4-6)	5 (4-6)	4 (3-6)	.23

Abbreviations: BMI, body mass index (calculated as weight in kilograms divided by height in meters squared); CHF, congestive heart failure; COPD, chronic obstructive pulmonary disease; MRSA, methicillin-resistant *Staphylococcus aureus*; qSOFA, quick Sequential Organ Failure Assessment; SIRS, systemic inflammatory response syndrome.

^a^ Due to the volume of cases, the Michigan Hospital Medicine Safety Consortium reports Black, White, and Other. Other includes the following categories: American Indian or Alaska Native, Arab or Chaldean ancestries, Asian, Native Hawaiian or Pacific Islander, and other (indicating that the patient is a race other than what is listed). A definition of how the Michigan Hospital Medicine Safety Consortium captures race information is included in the eAppendix in Supplement 1.

^b^ Any cancer includes, but is not limited to, malignant brain tumors, hematologic cancers, lymphoma, leukemia, lung cancer (small cell or non–small cell), ovarian cancer, colon cancer, prostate cancer, stomach/gastric cancer, pancreas/pancreatic cancer, kidney cancer, breast cancer, rectal/rectum cancer, bladder cancer, melanoma, liver cancer, uterine cancer, and metastatic cancer. This category does not include basal cell carcinoma, nonmelanoma skin cancer, squamous cell skin cancer, and inflammatory myofibroblastic pseudotumor without mention of cancer.

^c^ Immunosuppression includes chemotherapy administered within 30 days, HIV positive with a CD4 count greater than 200 cells/mm^3^, receiving a prednisone dose of 10 mg/d or more for at least 30 days (or equivalent corticosteroid dose), receiving biologic agents (eg, tumor necrosis factor inhibitors or other immunosuppressant agents), or congenital or acquired immunodeficiency.

^d^ Among those in whom procalcitonin testing was obtained.

^e^ Includes human metapneumovirus polymerase chain reaction, respiratory syncytial virus polymerase chain reaction or nucleic acid amplification, comprehensive viral respiratory screen, parainfluenza polymerase chain reaction, COVID-19 polymerase chain reaction, human rhinovirus/enterovirus polymerase chain reaction, and adenovirus polymerase chain reaction. Excludes influenza.

### Antibiotic Use in Patients Inappropriately Diagnosed With CAP

Patients inappropriately diagnosed with CAP received a median (IQR) of 7.0 (5.0-9.0) days of antibiotics, including 4.0 (3.0-5.0) inpatient days. The majority of inappropriately diagnosed patients (1821 of 2079 [87.6%]) received a full course of antibiotics. On bivariable analysis, patients treated with full vs brief, empirical antibiotics were more likely to be White (75.0% vs 66.7%; *P* = .004), have a history of COPD (44.6% vs 35.3%; *P* = .005), or present with a concurrent COPD exacerbation (28.2% vs 15.1%; *P* < .001) (eTable 3 in Supplement 1). On multivariable analysis, concurrent COPD exacerbation was associated with a full vs brief course of antibiotics (AOR, 1.74; 95% CI, 1.13-2.68; *P* = .01), while hemodialysis and negative procalcitonin (vs those not tested) were associated with brief courses (AOR, 0.29; 95% CI, 0.20-0.41; *P* < .001; and AOR, 0.47; 95% CI, 0.32-0.68; *P* < .001, respectively) (eTable 4 in (Supplement 1).

### Outcomes Associated With Full Course vs Brief Empirical Antibiotic Therapy Among Patients Inappropriately Diagnosed With CAP

The composite outcome of any 30-day postdischarge adverse event occurred in 536 of 2079 patients (25.8%) inappropriately diagnosed with CAP. Full vs brief empirical antibiotic therapy in patients inappropriately diagnosed with CAP was not associated with 30-day composite outcomes in either unadjusted or adjusted analyses (AOR, 1.00; 95% CI, 0.78-1.29; *P* = .99; Table 2). For individual 30-day outcomes, only physician-documented antibiotic-associated adverse events were associated with full vs brief therapy on unadjusted (31 of 1821 [2.1%] vs 1 of 258 [0.4%]) and adjusted (AOR, 7.23; 95% CI, 1.18-44.35; *P* = .03) analyses.

## Discussion

We applied a novel, validated metric to a unique dataset of manually collected data from hospitalized patients treated for CAP and found that approximately 1 in 8 patients were inappropriately diagnosed. Most hospitals inappropriately diagnosed more than 10% of patients. Patients at highest risk of inappropriate diagnosis were older, had dementia, or presented with altered mental status. Overall, nearly 88% of patients inappropriately diagnosed with CAP received a full antibiotic course, which was associated with physician-documented antibiotic-associated adverse events.

Why might physicians inaccurately diagnose CAP? First, because CAP is common, physicians are at high risk for cognitive biases such as availability bias (ie, the tendency to make decisions based on information that comes most readily to mind).^23,24^ Second, CAP symptoms are nonspecific and may overlap with other cardiopulmonary diseases (eg, congestive heart failure exacerbation), making diagnosis difficult. Given poor outcomes associated with CAP, in the setting of uncertainty, health care professionals may favor overtreatment rather than potentially missing a CAP diagnosis.^25,26,27^ Third, historical quality metrics imposed by organizations such as The Joint Commission (eg, requiring antibiotics within 6 hours of presentation) may have unintentionally led to more inappropriate diagnoses of CAP.^28,29^ These measures, in place in the 2000s and 2010s, may continue to affect health care professional practice behaviors related to diagnosis. Finally, previously published data show a correlation between inappropriate diagnosis of CAP and inappropriate diagnosis of urinary tract infection at the hospital level,^6^ suggesting that local policies, procedures, or culture may affect accurate diagnosis.

Unsurprisingly, we found that older patients, particularly those presenting with impaired cognition, had higher odds of inappropriate diagnosis. In one study of more than 45 000 patients, the rate of CAP ranged from 18.2 cases per 1000 patient-years among those aged 65 to 69 years to 52.3 cases per 1000 patient-years among those 85 years and older.^30^ The high underlying prevalence of CAP in older populations likely fuels previously discussed cognitive biases. Additionally, patients with cognitive impairment may have difficulty communicating. As a result, physicians may anchor on nonspecific data (eg, white blood cell count, fever in isolation) to make the diagnosis of CAP. Older adults—particularly those with dementia or altered mental status—are also more likely to be inappropriately diagnosed with other conditions, such as urinary tract infection (ie, asymptomatic bacteriuria).^31^ While altered mental status may be a sign of infection, including severe infection, it has a broad differential diagnosis (eg, polypharmacy, pain, dehydration), and anchoring on CAP may delay proper diagnosis and management. Finally, because older patients with CAP typically experience worse health outcomes,^32^ there may be an increased tendency to rapidly diagnose and treat presumed CAP, leading to higher rates of inappropriate diagnosis.

For patients at high risk of poor outcomes from delayed treatment of CAP, it may be pertinent to empirically prescribe antibiotics while finishing diagnostic evaluation. In these populations, guidelines recommend reconsideration, de-escalation, and cessation of antibiotics within 48 to 72 hours once infection has been ruled out.^20,21^ In the present study, we found little evidence of antibiotic cessation. Rather, patients empirically receiving antibiotic therapy for presumed CAP typically received a full antibiotic course. Compared with brief empirical antibiotics, we found that full courses were associated with antibiotic-associated adverse events. Making causal inferences about this association is complicated by the fact that length of treatment is not a baseline characteristic at the time of inappropriate diagnosis but a postdiagnosis observation and is thus endogenous (or likely to be determined in part by other predictors in the model). Nevertheless, longer durations of antibiotics are known to be associated with increased morbidity^9^ and delayed diagnosis of underlying conditions. Particularly, older patients who may have more comorbid diseases, or who are more likely to be taking medications that interact with antibiotics, are at high risk of harm from antibiotics and delayed diagnosis.^33,34,35^

### Strengths and Limitations

This study has limitations. First, because the assessment of diagnostic error relied on medical record review with limitations associated with documentation of alternative causes for signs, symptoms, or radiographic findings, we likely underestimate inappropriate diagnosis of CAP. Including inappropriately diagnosed patients within the CAP group may bias the risk factor analysis toward the null. Also, omissions in documentation of patient-documented symptoms could have resulted in misclassification of patients with appropriate diagnosis of CAP as inappropriate.^36,37^ Second, as mentioned previously, the finding that duration was associated with physician-documented antibiotic-associated adverse events does not prove causation, and, because few patients received brief antibiotic durations, the statistical power was limited, resulting in wide confidence intervals. Furthermore, documentation of antibiotic-associated adverse events may have influenced outcomes by lack of physician documentation or lack of attribution of symptoms to antibiotics, especially for those receiving a short duration of therapy. Third, bias from unmeasured confounders may exist. Fourth, we were unable to assess outcomes related to missed or delayed diagnosis or capture alternative diagnoses for those inappropriately diagnosed. Fifth, the ideal acceptable amount of inappropriate diagnosis of CAP is unclear. As the metric was designed to favor specificity over sensitivity (ie, ensuring a case classified as “inappropriate diagnosis” was inappropriately diagnosed), we believe that the proportion of patients inappropriately diagnosed should be close to, but not exactly, zero. Finally, diagnostic criteria for nonspecific CAP and findings such as fatigue or altered mental status may be attributed as symptoms of CAP. Although we used the best definition of CAP available, and included many objective criteria, inability of patients with altered mental status to convey symptom data remains a limitation.

This study also has several strengths. First, the diversity of hospitals improves generalizability. Second, abstractors underwent extensive, centralized training to ensure standardized data collection, and data were audited at routine intervals to ensure data validity. Third, the definition of inappropriate diagnosis was tested and subsequently endorsed by the NQF, increasing the validity of findings.

## Conclusions

This cohort study has important clinical and policy implications. Because hospitalizations for CAP are common, so too are inappropriate diagnoses of CAP. Risks of inappropriate diagnosis are not uniform across populations—already highly vulnerable groups are at highest risk of inappropriate diagnosis. These same vulnerable populations are also most likely to be affected by antibiotic-associated adverse events and resulting morbidity. Thus, balancing harms of underdiagnosis and overdiagnosis of CAP remains essential.

## Notes

Supplement 1.
eAppendix
**eTable 1.** Patient Flow **eTable 2.** Hospital Characteristics, n=48 **eTable 3.** Characteristics of Patients Treated for Community-Acquired Pneumonia by Antibiotic Duration, bivariable analysis **eTable 4.** Characteristics Associated with Full Course Antibiotic Therapy vs. Brief, Empiric Therapy among those Inappropriately Diagnosed with Community-Acquired Pneumonia, multivariable model

Supplement 2. Data Sharing Statement
