# Supplementary material for: Inappropriate Diagnosis of Pneumonia Among Hospitalized Adults
Source: JAMA Intern Med. 2024 Mar 25;184(5):548–56. doi: 10.1001/jamainternmed.2024.0077 (PMC10964165; doi:10.1001/jamainternmed.2024.0077)
Supplement: Supplement 1. — eAppendix eTable 1. Patient Flow eTable 2. Hospital Characteristics, n=48 eTable 3. Characteristics of Patients Treated for Community-Acquired Pneumonia by Antibiotic Duration, bivariable analysis eTable 4. Characteristics Associated with Full Course Antibiotic Therapy vs. Brief, Empiric Therapy among those Inappropriately Diagnosed with Community-Acquired Pneumonia, multivariable model [file jamainternmed-e240077-s001.pdf]

Supplemental Online Content

Gupta AB, Flanders SA, Petty LA, et al. Inappropriate diagnosis of pneumonia among hospitalized adults in Michigan. *JAMA Intern Med*. Published online March 25, 2024.
doi:10.1001/jamainternmed.2024.0077

eAppendix

eTable 1. Patient Flow

eTable 2. Hospital Characteristics, n=48

eTable 3. Characteristics of Patients Treated for Community-Acquired Pneumonia by Antibiotic Duration, bivariable analysis

eTable 4. Characteristics Associated with Full Course Antibiotic Therapy vs. Brief, Empiric Therapy among those Inappropriately Diagnosed with Community-Acquired Pneumonia, multivariable model

This supplemental material has been provided by the authors to give readers additional information about their work.

© 2024 Gupta AB et al. *JAMA Internal Medicine.*

eAppendix

Measure Specifications:

Question: *State the numerator.*

The measure quantifies adult, hospitalized medical patients inappropriately diagnosed with pneumonia. Here, inappropriate diagnosis is defined as patients treated with antibiotics for CAP who do not meet diagnostic criteria for pneumonia. Patients are considered inappropriately diagnosed if they did not have 2 or more signs or symptoms of pneumonia (documented at some point in the 2 days prior to the hospital encounter through the first 2 days of the hospital encounter) AND meet radiographic criteria for pneumonia.

Question: *Provide details needed to calculate the numerator.*

Patients in the numerator include those that did not have a) ≥2 signs or symptoms of pneumonia (documented at some point in the 2 days prior to the hospital encounter through the first 2 days of the hospital encounter) or did not b) meet radiographic criteria for pneumonia.

- Minor numerator exclusions:
  - Those whose only antibiotic treatment was azithromycin or doxycycline[a] (treatment could be related to chronic obstructive pulmonary disease exacerbation
- Excluded in NQF definition; considered appropriate diagnosis of pneumonia for manuscript
  - Those with a blood culture positive for a pathogenic bacteria
  - Those with a urine antigen positive for streptococcus or legionella

Signs and symptoms of pneumonia included cough, sputum production, dyspnea or tachypnea (respiratory rate >20 breaths/minute), hypoxemia (oxygen saturation <90% or partial pressure of arterial oxygen <60 mmHg), fever (temperature >38° Celsius) or hypothermia (≤36° Celsius), exam consistent with pneumonia (i.e., rales, crackles, dullness on percussion, bronchial breath sounds, or egophony), or Leukocyte count <4,000/µL; >10,000/µL; or >15% bands. Any combination of 2 or more symptoms is required to be considered appropriately diagnosed. Any patient who has 0 or 1 eligible signs or symptoms is considered inappropriately diagnosed with CAP and placed in the numerator.

In addition to signs and symptoms, data abstractors are instructed to review the medical record for any chest X-rays, chest computerized tomography (CTs), or abdominal CTs with lung findings to capture language that may be relevant to pneumonia (see table below). Chest x-rays, chest CTs, and abdominal CTs that are obtained in the 2 days prior to the hospital encounter through day 4 of the hospital encounter should be included.

Based on descriptions of radiographic criteria identified by abstractors, the following logic is used to determine if the patient met radiographic criteria for CAP for each individual image.

- Highest/first priority radiographic descriptions:
  - If interval improvement/resolution, no change from previous/no interval change, normal/no abnormalities or no evidence of pneumonia is documented→image considered NOT to meet radiographic criteria
- Second priority radiographic descriptions (overrides other findings except first priority, above):

© 2024 Gupta AB et al. *JAMA Internal Medicine.*

    - If air space density/opacity/disease, bronchopneumonia, cannot rule out pneumonia, cavitation, infection (cannot rule out infection/likely infection), infiltrate (any lobe specifications), loculations, pneumonia, necrotizing pneumonia, post-obstructive pneumonia, or consolidation is documented, then image considered to meet radiographic criteria
- If none of the above:
    - If ground glass is listed, then image considered to meet radiographic criteria
        - Exception: if ground glass plus interstitial lung disease, pulmonary edema or pulmonary vascular congestion is documented, then image considered NOT to meet radiographic criteria
    - If mass is listed, then image considered to meet radiographic criteria
        - Exception: If neoplasm/metastatic disease/malignancy is documented, then image considered NOT to meet radiographic criteria
    - If nodular air space disease, then image considered to meet radiographic criteria
        - Exception: If neoplasm/metastatic disease/malignancy or interstitial lung disease is documented, then image considered NOT to meet radiographic criteria
    - If pleural effusion, then image considered to meet radiographic criteria
        - Exception: If pulmonary edema, pulmonary vascular congestion, or ground glass is documented, then image considered NOT to meet radiographic criteria
    - If aspiration pneumonia, then image considered to meet radiographic criteria
        - Exception: If pneumonitis is documented, then image considered NOT to meet radiographic criteria

If there were multiple radiographic images, the following prioritization applies:

If available, chest CTs that occur within 1 calendar day (-1,0,+1) of a chest X-ray or abdominal CT are prioritized (even if they conflict with other results)

- If patient has any Chest CT meeting radiographic criteria, then patient considered to meet radiographic criteria
- If the patient's Chest CT does NOT meet radiographic criteria, then the patient is considered NOT to meet radiographic criteria, and then considered inappropriately diagnosed, add to numerator
- Example
    - Chest X-ray and Chest CT on day 1. Chest X-ray says pneumonia. Chest CT says no pneumonia. Patient considered inappropriately diagnosed.
    - Chest X-ray on day 1. Chest CT on day 5. Chest X-ray says pneumonia. Chest CT says no pneumonia. Patient not considered inappropriately diagnosed.

If no chest CT is present, the following applies

- If Abdominal CT AND/OR Chest X-Ray meet radiographic criteria, then patient considered to meet radiographic criteria
- If NEITHER Abdominal CT or Chest X-Ray meet radiographic criteria, then patient considered NOT to meet radiographic criteria, and considered inappropriately diagnosed, add to numerator

© 2024 Gupta AB et al. *JAMA Internal Medicine.*

Question: *State the denominator.*

The denominator includes all adult, general care, immunocompetent, medical patients hospitalized and treated for CAP who do not have a concomitant infection.

[a]Patients with COPD exacerbation treated with doxycycline alone excluded from manuscript, though not NQF measure, based on manuscript reviewer feedback.

Radiographic findings:

Radiographic Findings-Were any of the following documented? INCLUDE: All differentials recorded in the radiologist report AND provider documentation of image findings in provider notes Example: If X-Ray report reads "opacity in the right middle lobe, could be secondary to an asymmetric pulmonary edema versus pneumonia, select "Air Spaced Density/Opacity/Disease", "Pulmonary Edema" and "Pneumonia".
"*Abscess*" if the medical record indicates abscess in the image results.
"*Air Space Density/Opacity/Disease*" if the medical record indicates air space density, air space opacity or airspace disease in the image results. Example: Select if X-ray interpretation reads "numerous patchy areas of ground glass opacities."
"*Aspiration*" if the medical record indicates aspiration in the image results.
"*Aspiration Pneumonia*" if the medical record indicates aspiration pneumonia in the image results.
"*Bronchial wall thickening/pleural thickening*" if the medical record indicates bronchial wall thickening/pleural thickening in the image results. INCLUDE: Interstitial thickening, septal thickening
"*Bronchiectasis*" if the medical record indicates bronchiectasis in the image results. INCLUDE: Bronchiectactic changes
"*Bronchopneumonia*" if the medical record indicates bronchopneumonia in the image results.
"*Cannot Rule Out Pneumonia*" if the medical record indicates that pneumonia cannot be ruled out in the image results.
"*Cavitation*" if the medical record indicates cavitation in the image results. Example: Select if X-Ray interpretation states "Bilateral tiny ill-defined cavitary and noncavitary lung nodules."
"*Consolidation*" if the medical record indicates consolidation in the image results.
"*Emphysema/Emphysematous changes*" if the medical record indicates emphysema/emphysematous changes in the image results.
"*Granuloma*" if the medical record indicates granuloma(s) in the image results.
"*Ground Glass*" if the medical record indicates ground glass in the image results. INCLUDE: Documentation of ground glass as an individual finding.
"*Hyperinflation*" if the medical record indicates hyperinflation in the image results.
"*Infection (cannot rule out infection, likely infection)*" if the medical record indicates infection/cannot rule out infection/likely infection in the image results
"*Infiltrate (Single Lobe)*" if the medical record indicates infiltrate (single lobe) in the Image results.
"*Infiltrate (Multiple Lobes)*" if the medical record indicates infiltrate (multiple lobes) in the Image results.
"*Interstitial lung disease/interstitial disease*" if the medical record indicates interstitial lung disease/interstitial disease in the Image results. INCLUDE: Documentation of prominent interstitial lung markings, interstitial prominence, interstitial thickening.
"*Interval improvement or resolution*" if the medical record indicates interval improvement or resolution in the Image results.
"*Loculations*" if the medical record indicates loculations in the Image results.

© 2024 Gupta AB et al. *JAMA Internal Medicine.*

“*Mass*” if the medical record indicates mass in the Image results.
“*Mucus Plugging/Plugging*” if the medical record indicates mucus plugging/plugging in the Image results. *Note*: This is specific to Mucus Plugging/Plugging and not the term Mucus alone.
“*Necrotizing Pneumonia*” if the medical record indicates necrotizing pneumonia in the Image results.
“*Neoplasm/Metastatic Disease/Malignancy*” if the medical record indicates neoplasm/metastatic disease/malignancy in the Image results.
“*New or Worsening Infiltrates*” if the medical record indicates new or worsening infiltrates in the Image results.
“*Nodular Airspace Disease*” if the medical record indicates nodular airspace disease in the Image results.
“*Nodules”* if the medical record indicates nodules in the Image results. *Note*: Documentation of “nodes” does not equal nodules. Include: reticulonodular pattern Exclude: prominent mediastinal nodes
“*Pleural Effusion*” if the medical record indicates pleural effusion in the Image results.
“*Pneumonia*” if the medical record indicates pneumonia in the Image results. INCLUDE: Documentation in the report indicating concern for pneumonia, suggestive of pneumonia, pneumonia vs. post-obstructive process, nonspecific findings favoring focal pneumonia, suspicious for pneumonia, rule out (r/o) pneumonia, bilateral pneumonia is not excluded.
“*Pneumonitis*” if the medical record indicates pneumonitis in the Image results.
“Post Obstructive Pneumonia” if the medical record indicates post obstructive pneumonia in the Image results.
“*Pulmonary Edema*” if the medical record indicates pulmonary edema in the Image results.
“*Pulmonary Vascular Congestion*” if the medical record indicates pulmonary vascular congestion in the Image results. INCLUDE: pulmonary vascular markings, pulmonary vascular prominence, pulmonary vessels distended
“*No Evidence of Pneumonia*” if the medical record indicates no evidence of pneumonia in the Image results.
“*No Change from Previous/No Interval Change*” if the medical record indicates no changes from previous or no interval change in the Image results.
“*Normal/No Abnormalities*” if the medical record indicates the Chest/Abdominal CT is normal or no abnormalities were noted in the Image results.
“*None of the above statements*” if the medical record indicates something other than is listed above in the Image results.

© 2024 Gupta AB et al. *JAMA Internal Medicine.*

General Criteria for Exclusion from Michigan Hospital Medicine Safety (HMS) Collaborative Cohort

- Patient admitted initially to the intensive care unit or required mechanical ventilation
- Documentation that the patient was treated for an additional infection unrelated to pneumonia during hospitalization
- Patient severely immunocompromised:
    - Patient has any history of a heart, liver, lung, pancreas, bone marrow, stem cell, or hematopoietic stem cell transplant
    - Patient has a history of a kidney transplant within the year prior to the hospital encounter
    - Patient received treatment for the rejection of a kidney transplant in the 6 months prior to the hospital encounter
    - Patient has a diagnosis of Human Immunodeficiency Virus with a CD4 count <200 cells/µL on day 1 or day 2 of the hospital encounter or within 90 days prior to the hospital encounter
    - Patient has neutropenia (absolute neutrophil count <500 cells/µL) on day 1 or day 2 of the hospital encounter
- Patient is pregnant
- Patient admitted for comfort measures
- Patient left hospital against medical advice

© 2024 Gupta AB et al. *JAMA Internal Medicine.*

Michigan Hospital Medicine Safety (HMS) Collaborative
Pneumonia Data Elements Summary by Category

Enrollment Record (Required form)

- Demographics
  - Date of birth
  - Zip Code
  - Sex
  - Race/ethnicity
  - Insurance payor

Baseline (Required form)

- Admission Detail
  - Date of admission
  - Reason for admission
  - Admission source (e.g., emergency room, observation unit, direct admission, transfer)
  - Classification of attending physician at the time of admission
- Medications
  - Steroids/corticosteroids/immunosuppressive treatment (day #1 or day #2 of encounter or in 30 days prior to encounter)
  - Proton pump inhibitor (day #1 or day #2 of hospital encounter)
  - Histamine H2 Antagonist (day #1 or day #2 of hospital encounter)
- Co-Morbid Conditions
  - Acquired immune deficiency syndrome (AIDS)/Human immunodeficiency virus (HIV)
  - Cardiovascular disease
  - Cerebrovascular disease
  - COPD (Chronic Obstructive Pulmonary Disease)
  - Congestive Heart Failure (CHF)/Cardiomyopathy
  - Dementia
  - Diabetes (complicated or uncomplicated)
  - GERD
  - Hemiplegia
  - Inflammatory Bowel Disease
  - Mild, moderate, or severe liver disease
  - Moderate or severe kidney disease
  - Myocardial Infarction
  - Peptic ulcer disease
  - Peripheral vascular disorders
  - Rheumatoid Arthritis or Related Arthropathy/Connective Tissue Disease
  - Sickle Cell
- Physical Findings on Admission
  - Height/weight/Body mass index
  - Presence of swollen legs
  - Existing indwelling central venous catheter (CVC) on admission (inserted *prior* to admission)
  - Presence of a CVC that was inserted during this admission
- Social History
  - History of alcohol abuse (never user, current user, former user)
  - History of intravenous (IV) drug abuse (never user, current user, former user)

© 2024 Gupta AB et al. *JAMA Internal Medicine.*

- History of tobacco use (never user, current user, former user)

- Medical History
  - History of cancer (and type of cancer, if appropriate)
  - Type of cancer treatment (e.g., chemotherapy, hormonal therapy, surgical therapy, hospitalization related to cancer diagnosis) – if applicable
  - History of asplenia
  - History of congenital or acquired immunodeficiency
  - History of sepsis/Systemic inflammatory response syndrome (SIRS)/septic shock
  - History of Methicillin-resistant staphylococcus aureus (MRSA) infection
  - History of documented aortic aneurysm, aortic dissection, aortic rupture, or aortic repair
- Other History
  - Inpatient hospitalization for any reason in the 90 days prior to hospital encounter
  - Receipt of and type of intravenous therapy (IV antibiotics, chemotherapy, total parenteral nutrition (TPN), other)
  - Receipt of hemodialysis in the 30 days prior to the hospital encounter
  - Admission to (or resided in) nursing home or transferred from another hospital in the 30 days prior to the hospital encounter
  - On home oxygen (and how much)
- Laboratory
  - Albumin level on day #1 or day #2 of hospital encounter – if available
- Antibiotic History
  - Did patient receive an antibiotic in the 90 days prior to the hospital encounter
    - Name of antibiotic
    - Timeframe of administration (unknown, 1 to 30 days, 31 to 60 days, 61 to 90 days)
- Mobility
  - Fracture of hip/pelvis/leg on admission or in 30 days prior to admission
  - Presence of immobilizing cast
  - Paralysis on day #1 or day #2 of admission
  - Spinal cord injury with paralysis on admission or within the 30 days prior to admission
  - Trauma requiring hospitalization on admission or within the 30 days prior to admission
  - Immobilization >72 hours due to bed rest/paralysis
  - Active order for bed rest on day #1 or day #2 of admission
  - Braden activity score(s) of Bedfast or Chairfast on day #1 or day #2 of admission
- Antimicrobial Allergies (Present on Admission)
  - Medical record documents allergy to antibiotic prior to the hospital encounter (Emergency Department (ED), Observation, Inpatient)
    - Antibiotic Name
    - Reaction (e.g., anaphylaxis, diarrhea, fever, hives, itching, myalgias, nausea, etc.)
  - Contraindication (e.g., aortic aneurysm, myasthenia gravis, neuropathy, prolonged QT interval, etc.)

Antibiotics

- Antibiotic Administration
  - Name of antibiotic
  - Order date
  - Frequency and dose
  - Type of ordering physician

© 2024 Gupta AB et al. *JAMA Internal Medicine.*

- Indication for antibiotic documented in initial order (e.g., pneumonia, urinary tract infection, sepsis, etc.)
    - Primary medical team documented antibiotic indication in progress notes (day before antibiotic order, day of antibiotic order, day after antibiotic order)
- Antibiotic Discontinuation
    - Labs from day of discontinuation (if not continued through day of discharge or beyond)
    - Renal failure indicated on day of antibiotic discontinuation (during hospital encounter)
        - Was it reason for discontinuation
    - Rash indicated on day of antibiotic discontinuation (during hospital encounter)
        - Was it reason for discontinuation
    - Indicated reason for antibiotic discontinuation (day before discontinuation, day of discontinuation, day after discontinuation)

Culture

- Date of culture
- Type of culture (i.e., blood, sputum, etc.)
- Results (positive/negative)
    - Pathogen name (if positive
- Documentation of contaminated result
- Documentation of "normal flora" or "oral flora"
- Culture growth quantity
- Sensitivity analysis for pathogen

Discharge

- General information
    - Discharge date
    - Patient status (discharge, transfer, death)
        - Reason for intensive care unit (ICU) transfer for pneumonia (if applicable)
        - Date and cause of death (if applicable)
    - Discharge disposition (i.e., home, skilled nursing facility, etc.)
    - Primary and secondary diagnosis at time of discharge/transfer/death
- Discharge labs
    - Highest creatinine value
    - Lowest white blood cell count
    - Lowest platelet count
    - Lowest absolute neutrophil count
- Antibiotic Use
    - Anticipated duration of total antibiotic therapy (as documented in the patient's discharge summary)
    - Antibiotics prescribed at discharge
        - Name
        - Route (IV, Per Os (PO), etc.)
        - Duration/number of days of therapy
        - Dose and frequency of discharge antibiotic
        - Need for intravenous line for therapy

30-day Follow Up (Medical Record Review)

© 2024 Gupta AB et al. *JAMA Internal Medicine.*

- Encounter(s) with medical system (inpatient stay, ED visit, outpatient visit, observation visit)
    - Date of visit
- Indication for reason for hospitalization(s), ED visit(s), outpatient visit, observation visit
    - Adverse drug (antibiotic) event
    - Bacteremia
    - Clostridioides difficile (C. Diff)
    - COPD exacerbation
    - Pneumonia
    - Respiratory infection
    - Sepsis
    - Skin/soft tissue infection
    - Urinary tract infection
    - Unknown
- Documentation of an allergic reaction or adverse event related to an antibiotic in the 30 days following the hospital encounter
- Diagnosis of C. Diff infection in the 30 days following the hospital encounter
- Status of patient at the 30th day post-discharge

30-day follow-up phone call

- Number of follow up phone calls conducted (up to 3)
- Was patient deceased at time of follow up
    - If yes, cause of death
- Diagnosis of the following during 30 days following hospital encounter
    - Pneumonia
    - C. Diff
    - Sepsis
    - Skin/soft tissue infection
    - Urinary tract infection
- Antibiotic side effects (hives, fever, itching, nausea, anaphylaxis, anemia, seizures, etc.)
    - Receipt of care for this reaction
    - Did physician change medication due to reaction
- Symptoms while on antibiotics (or in the week after)
    - Thrush
    - Vaginal yeast infection
    - Abdominal pain
    - GI distress, upset stomach, nausea
    - Vomiting
    - Diarrhea
    - Skin rash/hives
- Complete course of antibiotics (and if not, why?)
- Other hospitalizations not documented in chart review
    - Date of admission
    - Date of discharge

Antimicrobial Allergy/Adverse Events (During the hospital encounter and in the 30 days following discharge)

- Antibiotic Name

© 2024 Gupta AB et al. *JAMA Internal Medicine.*

- Reaction type
- Date of reaction

Daily Entry

- Highest/lowest recorded vital sign (by day)
  - Temperature
  - Heart rate
  - Respiratory rate
  - Blood pressure (systolic/diastolic)
- Lowest oxygen saturation (by day)
- Lowest partial pressure of arterial oxygen (by day)
- Highest/lowest FiO2 (by day)
- Documentation of decreased urine output (by day)
- Mental status change (by day)
- Ability to take oral medications (by day)
- On day of discharge:
  - Last vital signs collected
- Antibiotics administered and route of administration (by day)

Pneumonia (Medical History)

- History or present Illness/Physical Exam
  - Documentation of any of the below on Day #1 or Day #2
    - Apnea
    - Bronchial breath sounds
    - Chest pain
    - Chest wall retractions
    - Chills
    - Cough
    - Crackles
    - Dullness on percussion
    - Dyspnea/shortness of breath
    - Egophony
    - Fatigue
    - Fever
    - Hemoptysis
    - Hypotension (systolic blood pressure of less than 90 mmHg)
    - Hypoxemia/Hypoxia
    - Increased secretions/sputum production
    - Mental status change or functional decline
    - Muscle aches
    - Rales
    - Rhinorrhea/runny nose
    - Rhonchi
    - Rigors
    - Sore throat
    - Use of accessory muscles
    - Wheezing

© 2024 Gupta AB et al. *JAMA Internal Medicine.*

- Did patient have an infectious disease (ID) consult and date of consult
  - Did patient have a pulmonary consult and date of consult
- Labs
  - Arterial pH from Day #1 or Day #2 of hospital encounter
  - White blood count from Day #1 or Day #2 of hospital encounter
    - If more than one, indicate highest and lowest
  - Lowest hematocrit from Day #1 or Day #2 of hospital encounter
  - Lowest platelet count from Day #1 or Day #2 of hospital encounter
  - Lowest sodium (Na) from Day #1 or Day #2 of hospital encounter
  - Highest BUN (blood urea nitrogen) from Day #1 or Day #2 of hospital encounter
  - Highest creatinine from Day #1 or Day #2 of hospital encounter
  - Highest glucose from Day #1 or Day #2 of hospital encounter
  - Highest total bilirubin from Day #1 or Day #2 of hospital encounter
  - Highest lactic acid from Day #1 or Day #2 of hospital encounter
  - Highest INR from Day #1 or Day #2 of hospital encounter
  - Ordering/collection of a respiratory culture during the encounter
  - Documentation of systolic blood pressure of <100mm/Hg from Day #1 or Day #2 of hospital encounter
    - Receipt of IV fluids following systolic blood pressure of <100mm/Hg
    - Receipt of vasopressor following systolic blood pressure of <100mm/Hg
  - Presence of fever (>37.8C) during the hospital encounter
- Pneumonia diagnosis during the hospital encounter
  - On admission (Day #1 or Day #2 of the inpatient admission), indicate type of pneumonia detailed by inpatient admitting physician
  - Type of pneumonia that is detailed by the emergency room provider
  - Was patient taking an antibiotic at start of encounter
  - Documentation of any of the following during the hospital encounter
    - Lung abscess
    - Empyema (and how treated)
    - Parapneumonic effusion (and how treated)
    - Necrotizing pneumonia
    - Cavitary pneumonia
    - Aspiration pneumonia (and did patient have an esophagram barium swallow study or speech pathology evaluation)
    - Tracheostomy (trach)
    - Post obstructive pneumonia
  - At the time of discharge, was there documentation of:
    - COPD exacerbation
    - CHF exacerbation
- Pneumonia medical history
  - History of pneumonia
  - History of kidney transplant (more than one year prior to admission)
  - History of asthma
  - History of COPD (severity and presence of FEV1 value)
  - History of structural lung disease (bronchiectasis, pulmonary fibrosis, interstitial lung disease)
  - History of nephrotic syndrome
  - Received wound care in the 30 days prior to hospitalization

© 2024 Gupta AB et al. *JAMA Internal Medicine.*

    - Received IV therapy in 30 days prior to hospitalization
    - Had billed OR time in 30 days prior to hospitalization
    - Received a feeding tube or administration of medications through a gastric tube in 7 days prior to hospitalization or on day #1 or Day #2 of hospitalization
    - Medical documentation of influenza or influenza pneumonia during the hospital encounter or in 90 days prior to hospital encounter

Pneumonia Labs (Non-Culture)

- Type of lab (i.e., brain natriuretic peptide (BNP), Urine Legionella)
- Date of lab
- Lab value (i.e., BNP)/final result (positive/negative)
- Organism tested (Influenza A/B, Human Metapneumovirus, Human Rhinovirus – Enterovirus, etc.)

Gram Stain of Respiratory Secretions

- Date of result
- Type of lab (i.e., sputum/bronchoalveolar lavage)
- Indication of any of the following:
    - Gram negative rods
    - Gram Negative Diplococci
    - Gram Positive Cocci (specify clusters, chains, pairs, diplococci; few, moderate, many etc.)
    - > 10 Squamous Epithelial Cells
    - Unacceptable for Culture/Unfit for Culture
    - Specimen Consistent with Saliva
    - Specimen rejected
    - Oral flora
    - Other

Chest/Abdominal CT

- Date of exam
- Was it a chest or abdominal CT
- Who ordered CT
- Was IV contrast utilized for exam
- Findings documented on CT (abscess, mass, infiltrates, ground glass, consolidation, etc.)

Chest X-Ray

- Date of exam
- Who ordered X-Ray
- Was X-Ray 1 view or 2 views
- Items documented on X-Ray (abscess, mass, infiltrates, ground glass, consolidation, etc.)

© 2024 Gupta AB et al. *JAMA Internal Medicine.*

List of Included ICD-10 Codes

ICD10 Codes	ICD 10- Label
A48.1	Legionnaires' disease
J09.X1	Influenza due to ident novel influenza A virus w pneumonia
J10.00	Flu due to oth ident flu virus w unsp type of pneumonia
J10.01	Influenza due to other identified influenza virus with the same other identified influenza virus pneumonia
J10.08	Influenza due to oth ident influenza virus w oth pneumonia
J11.00	Flu due to unidentified flu virus w unsp type of pneumonia
J11.08	Flu due to unidentified flu virus w specified pneumonia
J12.0	Adenoviral pneumonia
J12.1	Respiratory syncytial virus pneumonia
J12.2	Parainfluenza virus pneumonia
J12.3	Human metapneumovirus pneumonia
J12.81	Pneumonia due to SARS-associated coronavirus
J12.89	Other viral pneumonia
J12.9	Viral pneumonia, unspecified
J13	Pneumonia due to Streptococcus pneumoniae
J14	Pneumonia due to Hemophilus influenzae
J15.0	Pneumonia due to Klebsiella pneumoniae
J15.1	Pneumonia due to Pseudomonas
J15.20	Pneumonia due to staphylococcus, unspecified
J15.211	Pneumonia due to methicillin suscep staph
J15.212	Pneumonia due to Methicillin resistant Staphylococcus aureus
J15.29	Pneumonia due to other staphylococcus
J15.3	Pneumonia due to streptococcus, group B
J15.4	Pneumonia due to other streptococci
J15.5	Pneumonia due to Escherichia coli
J15.6	Pneumonia due to other aerobic Gram-negative bacteria
J15.7	Pneumonia due to Mycoplasma pneumoniae
J15.8	Pneumonia due to other specified bacteria
J15.9	Unspecified bacterial pneumonia
J16.0	Chlamydial pneumonia
J16.8	Pneumonia due to other specified infectious organisms
J17	Pneumonia in diseases classified elsewhere
J18.0	Bronchopneumonia, unspecified organism
J18.1	Lobar pneumonia, unspecified organism
J18.2	Hypostatic pneumonia, unspecified organism
J18.8	Other pneumonia, unspecified organism
J18.9	Pneumonia, unspecified organism
J69.0	Pneumonitis due to inhalation of food and vomit

© 2024 Gupta AB et al. *JAMA Internal Medicine.*

J69.8	Pneumonitis due to inhalation of other solids and liquids
J84.111	Idiopathic interstitial pneumonia, not otherwise specified
J84.116	Cryptogenic organizing pneumonia
J84.117	Desquamative interstitial pneumonia
J84.2	Lymphoid interstitial pneumonia
J85.1	Abscess of lung with pneumonia
J85.2	Abscess of lung without pneumonia

© 2024 Gupta AB et al. *JAMA Internal Medicine.*

Michigan Hospital Medicine Safety (HMS) Collaborative
Definition of sex, race, ethnicity

Sex

Instructions: Review the medical record to determine the sex of the patient.
This is a required field and the form cannot be submitted without an entry in this field.
Select one of the following:

- *"Man"* if the patient is categorized as a man in the medical record.
- *"Woman"* if the patient is categorized as a woman in the medical record.
- *"Unknown"* if the patient's sex is unknown.

Ethnicity

Instructions: Review the medical record to determine the patient's ethnicity.
Select one of the following:

- *"Hispanic or Latino"* if patient demographic information indicates patient is of Hispanic descent. The US Census Bureau states that "People who identify their origin as Spanish, Hispanic, or Latino may be of any race."
- *"Non-Hispanic or Latino"* if patient demographic information indicates patient is not of Hispanic descent.
- "*Unknown*" if ethnicity is not reported in the medical record.

Race

Instructions: Review the medical record to determine the patient's race.
Select one of the following:

- *"American Indian or Alaskan Native"* if patient demographic information indicates patient is Native American, American Indian, or Alaska Native.
- *"Arab and Chaldean Ancestries"* if the patient demographic information indicate patient is of Arab or Chaldean Ancestries.
- *"Asian"* if patient demographic information indicates Asian.
- *"Black or African American"* if patient demographic information indicates patient is black or African American.
- *"Native Hawaiian or Pacific Islander"* if patient demographic information indicates patient is Native Hawaiian or Pacific Islander.
- *"White or Caucasian"* if patient demographic information indicates patient is white or Caucasian.
- *"Other"* if patient demographic information indicates the patient is a race other than what is listed above.
- *"Unknown"* if patient's race is not indicated in the medical record.

© 2024 Gupta AB et al. *JAMA Internal Medicine.*

Michigan Hospital Medicine Safety (HMS) Collaborative
Data Selection Criteria – Antimicrobial

Chart Abstraction

Data collection for the HMS Antimicrobial Use Initiative will involve the abstraction of patient data for 16 eligible cases every 2 weeks. A specific sampling process is employed for the project to prevent case selection bias. The process uses a 14-Day cycle that works as follows: The clinical data abstractor abstracts data on 16 eligible cases from discharge dates covered by a 2-week period. To minimize sampling bias, abstractors are expected to select cases from every day of the cycle being abstracted, if possible.

The HMS Antimicrobial Use Initiative focuses on two (2) infectious disease states: pneumonia & urinary tract infections. Eight (8) cases of each infectious disease state should be collected each cycle when possible. There are standard data entry forms that apply to both infectious disease states, in addition to infectious disease state-specific forms.

Sampling Procedure

The data collection for each eligible case will capture data from 1 year prior to the hospital encounter through 30 days following the hospital discharge.

To accomplish the selection of cases, the clinical data abstractor completes the following:

1. For each abstraction cycle, obtain a list of patients admitted to all medical services during the timeframe covered by the cycle. The HMS Coordinating Center creates a sample list to provide to the site's informatics/IT department. The list may or may not already have exclusion filters applied, depending on the capabilities of the hospital.
2. Organize the list chronologically by date and time of discharge. Cases to be abstracted will be from patient discharges 30 days prior.
3. Begin with the first patient on the chronological list and review the patient record to confirm that the patient should not be excluded. If excluded, document which exclusion criteria applied. If not excluded, the patient is eligible and data abstraction should occur.
4. Complete a 30-Day Follow-up for patients who are still alive at the end of the hospital stay and were not transferred to the intensive care unit (ICU) during the hospitalization of interest. (Data collection stops after a transfer to ICU occurs.)
5. Complete a 30-Day Phone Call for patients who are eligible for a phone call.
6. Repeat this process by continuing to review patients from the chronological list making sure that cases are distributed across all the days of the 14-day cycle to the greatest extent possible (meaning discharge dates across all days of the week Sunday-Saturday) until 16 eligible cases are identified and abstracted for each 2-week period.

© 2024 Gupta AB et al. *JAMA Internal Medicine.*

Michigan Hospital Medicine Safety (HMS) Collaborative
Data Curation Methods – General

Details on the HMS Databases

HMS has three (3) databases, or registries, currently in production: PICC/Midline, Antimicrobial Use (ABX), and Sepsis. The data in this manuscript is generated from the ABX database. The database is used as the primary hub of case abstraction, data reporting, case volume analysis, resource gathering and abstraction queries. The database uses both Drupal and LimeSurvey software for data collection/abstraction, which are maintained by the HMS Coordinating Center and our database administrative team. For data reporting, HMS uses Business Objects software to allow hospitals participating in HMS to access their updated data on a daily basis.

For resource gathering and abstraction queries, HMS utilizes a link to a Zendesk guide, which allows abstractors to submit questions regarding data abstraction/reporting, obtain updated data definitions and access resources and quality improvement tools.

Upgrades

Upgrades to the HMS databases occur at least once per year. Upgrades may occur more frequently, depending on updates made to the project throughout the year and changing data needs for quality improvement projects. During an upgrade, the HMS database undergoing updates is taken offline and is unavailable for data abstraction. Upgrades may occur if we have spelling or grammatical errors to fix, selections to add or remove, questions to add or remove, branching questions to add or remove, and/or functionalities to improve or update. After completion of an upgrade, data entered prior to the upgrade is archived and restored in the database. If new questions are added, the abstractors are not expected to return to previously entered cases to enter new data fields as case entry can span several years. The HMS Coordinating Center keeps track of all updates using a ticketing system and the data analytics and statistician team are made aware of all updates.

Data Validation: Audits

Audits are conducted to ensure that the data is being collected consistently across all participating hospitals. The goal is to identify issues with the abstraction process so that they can be appropriately addressed via education and/or changes to the data entry system. Each HMS-participating hospital is audited by a trained member of the Coordinating Center at least once per year. On average ~50 audits have been conducted per year since the launch of HMS in 2011. This number increases each year as new hospitals join the collaborative. It is the expectation that each audited site will attain a 95% or greater rate of accuracy to receive full points on the HMS Performance Index. To determine the audit score, the auditor calculates a score for each individual case based on the average number of audit fields as noted below (see Medical Record Review). Then using the individual scores for each case, an overall audit score is calculated by averaging all of the audit cases combined. If a site receives a score of less than 95% on an audit, every attempt will be made to re-audit that site in the same year.

The audit consists of four parts: medical record review, review of eligibility lists, review of inclusion/exclusion criteria and practices, and post-audit follow up.

© 2024 Gupta AB et al. *JAMA Internal Medicine.*

Medical Record Review:

The primary focus of the audit is a medical record review of pre-selected cases by one to three HMS auditors. For each initiative, key complication cases are required to be audited to ensure accurate outcome measures for reporting purposes. For example, in the ABX database, *Clostridioides* difficle is assessed. Prior to the audit, the primary auditor queries the data analytics team to obtain the list of required complication cases that are due for audit and a random sample of additional non-complication cases. On average 7 to 10 cases are audited if one auditor is present. If a site has a large number of unaudited complication cases, a second or third auditor will join to complete additional cases. The list of cases is distributed to the abstractor 1 to 2 weeks in advance of the audit. Prior to sending the list of audited cases, the abstractor is locked from making updates to previously completed cases. Upon the on-site audit, the auditor(s) independently reviews the medical documentation for each case from the hospital's Electronic Medical Record (EMR) and compares it to what was entered into the HMS database. At the end of the audit day, the auditor's case findings and discrepancies between the EMR and the information entered into the HMS databases (if applicable) will be reviewed in detail with the abstractor. At the resolution of the audit, these discrepancies (if validated as incorrect by both the abstractor and auditor), are corrected in the database by the abstractor to ensure case accuracy. The auditor will also provide additional education, as needed, as issues are identified. If during the medical record review a completed eligible case is determined by the auditor to be ineligible, a score of 90% is assigned to the case and added to the overall average score. The pneumonia cohort contains over 2,000 fields.

Eligibility List Review:

The second item reviewed during an audit is the eligibility/discharge lists and coding at the site being audited. Prior to the audit, the abstractor connects with their hospital's information technology (IT) group for the coding used to generate their eligibility/discharge lists for each project PICC/Midline (one list for both) and Antimicrobial (separate lists for Pneumonia and Positive Urine Culture). This coding is reviewed by the auditor and feedback is provided regarding updates that need to be made to the coding, if applicable.

Inclusion/Exclusion Criteria Review:

The final item reviewed during an audit is inclusion/exclusion criteria. The purpose of this review is to ensure that the abstractor understands the inclusion/exclusion criteria for each project and is applying those criteria appropriately when reviewing cases. At least one case for each project deemed ineligible by the abstractor is randomly selected and reviewed with the auditor(s). Once a case is identified, the abstractor shows the auditor(s), in the medical record, the reason the case was excluded from abstraction. If a case was deemed ineligible by the abstractor, but was determined through this review that it was actually eligible for abstraction, another case from the same project will be reviewed until a legitimate ineligible case is found. If the abstractor has incorrectly identified a case as ineligible, the auditor(s) will provide additional on-site education about eligibility criteria. If more than 2 randomly-selected cases were deemed ineligible by the abstractor, but are determined to be eligible for abstraction after review, a score of 90% will be added to the final audit summary for each additional case that is found to be eligible.

© 2024 Gupta AB et al. *JAMA Internal Medicine.*

Post-Audit Follow Up:

After the audit has concluded, the primary auditor composes a summary of the findings, including specific areas to update in the HMS databases, education provided to the site during the audit, and a summary of any findings from the eligibility/discharge list review. The final audit summary is provided to the site within two to three weeks of completion of the audit. This summary will be sent to the site's abstractor(s), quality administrator, and physician champion. The summary will include a percentage score for the audit, which is calculated based on the average of the scores for all cases reviewed. Upon receiving the final audit summary, the abstractor(s) has three months from the date of receipt to make all updates in the HMS database noted in the final report. The final audit score is then factored into the sites performance index scorecard for the given year. During a typical year, 5% of the performance index is associated with the audit score(s) completed during the performance year.

Data Validation: Data Checker

Each HMS database, including ABX, has a robust data checker that can identify in real time errors in abstraction that have occurred on a case-by-case basis. Abstractors are trained to run a data checker on each case before submitting it to the database so that any data errors are identified at the time of the initial abstraction and can be corrected prior to submission. Additionally, a live daily report is available, which provides a culmination of all data errors on all cases entered into the database that an abstractor is able to see in order to correct potential discrepancies in data abstraction.

The ABX database has a total of 226 individual checks. These data checks range from potential length of stay issues (i.e., a case where the length of stay = 1) to verification that cultures are appropriately entered (i.e., it was noted in a form that a respiratory culture was collected, but no respiratory culture form was completed for that case). The data checker is also utilized to highlight which days in our Daily Entry tab need to be completed for that case. As a note, the "Daily Entry tab" is the section of forms that are utilized to enter daily antibiotic and vital sign information for ABX cases. After the entry of all data forms, the abstractor runs the data checker for that case and upon completion of the data checker validation, the Daily Entry tab days that need to be completed are highlighted in red and all days that do not need to be completed remain gray. This allows the abstractors to know exactly which days need to be entered and verify that they have fully completed data abstraction for that case.

Data Validation: Global Data Checks

The HMS Coordinating Center conducts global data checks on an ad hoc basis during the data analysis process to identify any issues that might occur across the entire database that may not be included in the data checker. These global data checks are typically run when we identify an error as part of another process, such as coding a report and realizing something does not pull into the report as expected. The HMS Coordinating Center will do data queries to sites throughout the year with prompts to analyze their data in accordance with the medical record if we notice discrepancies outside of the data checker elements.

Data Validation: Site Specific

Each hospital receives site specific data reports via a printed version quarterly and daily within the database/registry. Included in these reports are the sites overall score for each measure by quarter and a detailed list of cases that have been identified as opportunities for improvement (i.e. also titled fall-

© 2024 Gupta AB et al. *JAMA Internal Medicine.*

outs). Each hospital is encouraged to review these fall-outs with their local team to perform audit and feedback, identify trends, and assist with overall quality improvement. Occasionally, during this review the local team will identify a potential issue with how the fall-out was determined based on the clinical scenario. In some instances, the case is reviewed and justification for the coding/calculation is reinforced to the local site. In other instances, modifications to the code and/or additional modifications to the data registry questions are required. Typically, the latter is more common at the initial launch of a new measure. For more longstanding measures, modifications are rare.

© 2024 Gupta AB et al. *JAMA Internal Medicine.*

eTable 1. Patient Flow

Criteria	Patients included	Patients removed
Total patients in the pneumonia cohort	19906	
Missing age or sex	19897	9
Hospitals with < 10 patients	19884	13
Antibiotic duration > 14 days	18855	1029
Azithromycin monotreatment[a]	18792	63
Doxycycline monotreatement[a]	18732	60
Received care in an intensive care unit	18383	349
Transferred to another hospital	18142	241
Died during hospitalization (missing antibiotic duration)	18016	126
Missing antibiotic duration for another reason	17290	726

[a]With concurrent exacerbation of chronic obstructive pulmonary disease (COPD)

© 2024 Gupta AB et al. *JAMA Internal Medicine.*

eTable 2. Hospital Characteristics, n=48

**Characteristic**	**Number (%)**
Teaching Hospital	36 (75.00)
Rurality[a]	
Metropolitan	9 (18.75)
Non-metropolitan	39 (81.25)
Bed Size	
<250 beds	18 (37.50)
250 – 500 beds	20 (41.67)
>500 beds	10 (20.83)
Hospital Profit Type	
Not for Profit	4 (8.33)
For Profit	44 (91.67)

[a]Rurality assessed based on Rural-Urban Continuum Codes (RUCCs), which are assigned at the county level. Counties with RUCCs <4 are considered metropolitan. Counties with RUCCs ≥4 are considered non-metropolitan.

Economic Research Service. U.S. Department of Agriculture. Rural-Urban Continuum Code (2013). Last updated 10 December 2020. <https://www.ers.usda.gov/data-products/rural-urban-continuum-codes/> Accessed 12 August 2023.

© 2024 Gupta AB et al. *JAMA Internal Medicine.*

eTable 3. Characteristics of Patients Treated for Community-Acquired Pneumonia by Antibiotic Duration, bivariable analysis

	Inappropriate Diagnosis of CAP (N=2079)	Antibiotic Duration		p value
		Brief, Empiric (N=258)	Full Course (N=1821)	
**Age**				0.42
Median (IQR)	71.8 (60.1-82.8)	70.9 (59.3-82.6)	71.9 (60.5-82.8)	
**Age group**				0.31
<65 years	715 (34.4%)	99 (38.4%)	616 (33.8%)	
65-74 years	463 (22.3%)	51 (19.8%)	412 (22.6%)	
≥ 75 years	901 (43.3%)	108 (41.9%)	793 (43.5%)	
**BMI**				
Median (IQR)	27.3 (23.1-32.8)	27.6 (23.5-34.0)	27.3 (23.0-32.5)	0.27
**Sex**				0.27
Men	1034 (49.7%)	120 (46.5%)	914 (50.2%)	
Women	1045 (50.3%)	138 (53.5%)	907 (49.8%)	
**Race**				0.01
Black	468 (22.6%)	73 (28.3%)	395 (21.8%)	
White	1534 (74.0%)	172 (66.7%)	1362 (75.0%)	
Other[a]	71 (3.4%)	13 (5.0%)	58 (3.2%)	
**Ethnicity**				0.22
Hispanic	46 (2.2%)	7 (2.7%)	39 (2.1%)	
Non-Hispanic	1666 (80.1%)	215 (83.3%)	1451 (79.7%)	
Unknown	367 (17.7%)	36 (14.0%)	331 (18.2%)	
**Insurance Type**				0.46
Commercial	560 (27.7%)	77 (30.8%)	483 (27.2%)	
Public	1443 (71.3%)	171 (68.4%)	1272 (71.7%)	
Self-Pay	22 (1.1%)	2 (0.8%)	20 (1.1%)	
**Pneumonia Severity Index**				
Median (IQR)	96.0 (74.4-119.3)	96.5 (76.4-122.6)	95.7 (74.0-118.4)	0.27
**qSOFA**				
Mean (SD)	0.72 (0.64)	0.75 (0.63)	0.72 (0.64)	0.39
Score ≥2	190 (9.1%)	25 (9.7%)	165 (9.1%)	0.74
Score <2	1889 (90.9%)	233 (90.3%)	1656 (90.9%)	

© 2024 Gupta AB et al. *JAMA Internal Medicine.*

	Inappropriate Diagnosis of CAP (N=2079)	Antibiotic Duration		p value
		Brief, Empiric (N=258)	Full Course (N=1821)	
**SIRS $\geq$2 and end organ dysfunction**	607 (29.2%)	87 (33.7%)	520 (28.6%)	0.09
**CHF Exacerbation**	152 (7.3%)	22 (8.5%)	130 (7.1%)	0.42
**COPD Exacerbation**	552 (26.6%)	39 (15.1%)	513 (28.2%)	<0.001
**Altered mental status**	223 (10.7%)	36 (14.0%)	187 (10.3%)	0.07
Altered mental status without dementia	134 (6.4%)	18 (7.0%)	116 (6.4%)	0.71
**Comorbidities**				
Charlson Comorbidity Index; median (IQR)	3.0 (1.0-4.0)	3 (1-5)	3 (1-4)	0.08
Influenza	67 (3.2%)	14 (5.4%)	53 (2.9%)	0.03
CHF	542 (26.1%)	71 (27.5%)	471 (25.9%)	0.57
COPD	904 (43.5%)	91 (35.3%)	813 (44.6%)	0.005
Diabetes	674 (32.4%)	95 (36.8%)	579 (31.8%)	0.11
Dementia	329 (15.8%)	45 (17.4%)	284 (15.6%)	0.45
CKD	561 (27.0%)	93 (36.1%)	468 (25.7%)	<0.001
Any Cancer	436 (21.0%)	52 (20.2%)	384 (21.1%)	0.73
Lung cancer[b]	79 (3.8%)	6 (2.3%)	73 (4.0%)	0.19
Current of former smoker	1331 (64.0%)	152 (58.9%)	1179 (64.7%)	0.07
Home oxygen	301 (14.5%)	24 (9.3%)	277 (15.2%)	0.01
Immunosuppression[c]	116 (5.6%)	13 (5.0%)	103 (5.7%)	0.69
History of MRSA infection	5 (0.2%)	1 (0.4%)	4 (0.2%)	0.49
**Procalcitonin Obtained**	437 (21.0%)	77 (29.8%)	360 (19.8%)	<0.001
**Procalcitonin Range[d]**				0.13
0-0.1	212 (48.5%)	46 (59.7%)	166 (46.1%)	
0.1-0.25	89 (20.4%)	15 (19.5%)	74 (20.6%)	
0.25-0.5	48 (11.0%)	6 (7.8%)	42 (11.7%)	
>0.5	88 (20.1%)	10 (13.0%)	78 (21.7%)	
Missing	1642	181	1461	
**Respiratory Viral Panel[e]**				0.02
Positive	47 (2.3%)	37 (2.0%)	10 (3.9%)	
Negative	291 (14.0%)	245 (13.5%)	46 (17.8%)	
No test/missing	1741 (83.7%)	1539 (84.5%)	202 (78.3%)	
**Hemodialysis within 30 days prior to encounter**	74 (3.6%)	24 (9.3%)	50 (2.7%)	<0.001

© 2024 Gupta AB et al. *JAMA Internal Medicine.*

	Inappropriate Diagnosis of CAP (N=2079)	Antibiotic Duration		p value
		Brief, Empiric (N=258)	Full Course (N=1821)	
**Antibiotics within the last 90 days**	465 (22.4%)	60 (23.3%)	405 (22.2%)	0.71
**Hospital admission in prior 90 days**	535 (25.7%)	67 (26.0%)	468 (25.7%)	0.93
**Admitted from outside skilled facility**	86 (4.1%)	7 (2.7%)	79 (4.3%)	0.22
**Admission from home, discharged to outside facility**	251 (12.1%)	30 (11.6%)	221 (12.1%)	0.81
**Functional Status on admission**				
Bedridden	82 (3.9%)	6 (2.3%)	76 (4.2%)	0.15
Wheelchair	111 (5.3%)	16 (6.2%)	95 (5.2%)	0.51
**Discharged to outside skilled facility**	470 (22.6%)	58 (22.5%)	412 (22.6%)	0.96
Skilled nursing facility	292 (14.0%)	39 (15.1%)	253 (13.9%)	0.60
Subacute rehabilitation	80 (3.8%)	11 (4.3%)	69 (3.8%)	0.71
Long-term care facility	5 (0.2%)	1 (0.4%)	4 (0.2%)	0.48
Acute rehabilitation facility	17 (0.8%)	2 (0.8%)	15 (0.8%)	0.99

Full course of antibiotics indicates >3 days of therapy; Brief, empiric course of antibiotics indicates $\leq$3 days of therapy

BMI= body measurement index; qSOFA= quick sequential organ failure assessment; SIRS= systemic inflammatory response syndrome criteria; CHF= congestive heart failure; COPD= chronic obstructive pulmonary disease; CKD= chronic kidney disease; MRSA= methicillin-resistant staphylococcus aureus; IQR= interquartile range; SD= standard deviation

[a] Due to volume of cases, HMS reports out white, black, and other. Other includes the following categories: American Indian or Alaskan Native, Arab or Chaldean Ancestries, Asian, Native Hawaiian or Pacific Islander and Other. A definition of how HMS captures race information is included in the Appendix.

[b] Any cancer includes (but not limited to): malignant brain tumors, hematologic malignancies, lymphoma, leukemia, lung cancer (small cell or non-small cell), ovarian cancer, colon cancer, prostate cancer, stomach/gastric cancer, pancreas/pancreatic cancer, kidney/renal cancer, breast cancer, rectal/rectum cancer, bladder cancer, melanoma, liver cancer, uterine cancer, metastatic cancer, etc. Does not include basal cell carcinoma, non-melanoma skin cancer, squamous cell skin cancer, inflammatory myofibroplastic pseudotumor without mention of malignancy

© 2024 Gupta AB et al. *JAMA Internal Medicine.*

[c] Chemotherapy administered within 30 days, Human Immunodeficiency Virus positive with a CD4 count greater than 200 cells/mm3 , prednisone dose of 10 mg/day or more for at least 30 days (or equivalent corticosteroid dose), receiving biologic agents (e.g., Tumor Necrosis Factor inhibitors or other immunosuppressant agents), or congenital or acquired immunodeficiency.

[d] Percentages are of those where procalcitonin testing was obtained

[e] Includes human metapneumovirus Polymerase chain reaction (PCR); Respiratory Syncytial Virus PCR or Nucleic acid amplification (NAA); Viral Respiratory Screen, Comprehensive; Parainfluenza PCR, Coronavirus PCR, Human rhinovirus/enterovirus PCR, adenovirus PCR. Excludes influenza

© 2024 Gupta AB et al. *JAMA Internal Medicine.*

eTable 4. Characteristics Associated with Full Course Antibiotic Therapy vs. Brief, Empiric Therapy among those Inappropriately Diagnosed with Community-Acquired Pneumonia, multivariable model

	aOR (95% CI)	P
Age (per additional 10 years)	1.02 (0.95-1.09)	0.61
Home Oxygen	1.32 (0.84-2.05)	0.23
COPD Exacerbation	1.74 (1.13-2.68)	0.01
Hemodialysis	0.29 (0.20-0.41)	<0.001
Procalcitonin (Negative)	0.47 (0.32-0.68)	<0.001
Procalcitonin (Positive)	1.18 (0.63-2.21)	0.61
Procalcitonin (Not tested)	REF	REF

aOR= Adjusted Odds Ratio; CI= Confidence Interval; COPD= Chronic Obstructive Pulmonary disease; Procalcitonin>0.25 was considered positive

© 2024 Gupta AB et al. *JAMA Internal Medicine.*
