# Supplementary material for: Inappropriate Diagnosis of Pneumonia Among Hospitalized Adults
Source: JAMA Intern Med. 2024 Mar 25;184(5):548–56. doi: 10.1001/jamainternmed.2024.0077 (PMC10964165; doi:10.1001/jamainternmed.2024.0077)
Supplement: Supplement 2. — Data Sharing Statement [file jamainternmed-e240077-s002.pdf]

Data Sharing Statement

Gupta. Inappropriate Diagnosis of Pneumonia Among Hospitalized Adults in Michigan. *JAMA Intern Med*. Published March 25, 2024. doi:10.1001/jamainternmed.2024.0077

Data
Data available: No

Additional Information
Explanation for why data not available: As our initiative is a collaborative of hospitals in Michigan focusing on improving the quality of care for hospitalized patients, we utilized the existing infrastructure of the Michigan Hospital Medicine Safety (HMS) Consortium. The data use agreements among the participating hospitals prohibit us from sharing our registry data outside of the Coordinating Center, given hospital specific performance is directly identifiable.
